# The Week's Work

**Published:** 1910-10-22

**Authors:** 


### The Week's Work.

### THE FIRE AT THE LEEDS INFIRMARY.

An old student of the Leeds Infirmary contributes to the *Yorkshire Post* a letter drawing attention to the fact that the recent fire which took place at the infirmary is almost certain to put the Committee to considerable expense, even though the loss is covered by insurance. He suggests, therefore, that a fund should be opened for the purpose of helping the Committee to deal with the matter of rebuilding. The suggestion is a good one, and we trust that it will commend itself to friends of the institution. As the writer of the letter justly remarks, the hospital is doing, and has in the past done, considerable good work, which has won for it many staunch friends in the district and a reputation outside Yorkshire. It is highly essential that that good work should not be stopped or interfered with in any way owing to financial considerations. The untoward event that has caused such damage should be the means of prompting all those who appreciate the good work of the institution to help the Committee to the utmost of their power.

### SICKNESS INSURANCE.

In the current issue of the *Charity Organisation Review* Dr. Loch writes on some pros and cons of invalidity insurance, his paper being practically an extension and revision of the address delivered before the British Medical Association in July last. It is highly necessary, especially at the present time, when the whole question of hospital relief is attracting so much attention, that institutional workers should be conversant with the details and the arguments for and against any scheme of invalidity insurance. In a recent number of The Hospital (October 1) we published an article from the pen of Dr. Bosscha, the Superintendent of the General Hospital at Utrecht, and one of the best-known hospital authorities on the Continent, in which was explained the immense influence which the German system of sickness and invalidity insurance has had upon German hospitals. The facts and figures given in that paper are both instructive and suggestive, and readers of Grotjahn's book, on which the summary was based, will readily admit that the important conclusions put forward by Dr. Bosscha were eminently fair. Dr. Loch's opinions are equally well worth consideration, although he approaches the subject from a slightly different point of view, and those interested in the question ought to pay careful attention to his paper, which is one of the best summaries on the subject that has appeared in an English journal.

### THE DIFFERENT ASPECTS.

In dealing with this question it is only fair to recognise that there are different interests to be considered. The three courses discussed by Dr. Loch are what he terms the discriminative economic contract on voluntary lines, the discriminative economic contract on compulsory lines, and the indiscriminative non-economic contract on compulsory lines. The objections to the first-mentioned method are obvious enough; equally so are the disadvantages of the third method, which practically amounts to indiscriminate gratuitous relief. It is the second method, then, which is virtually the method adopted in Germany that seems to promise salvation. Dr. Loch thinks that both the first and the second methods have features in common, and it is true enough that there is some family likeness between them in theory. In practice, however, they are utterly dissimilar, for the one depends on purely voluntary principles, while the other means the obligatory practice of thrift by putting aside certain portions of income for insurance purposes. Yet Dr. Loch appears to think that the dispensary system is preferable to the compulsory insurance system. "That a dispensary system would impose on the country a much lesser burden of administration than an insurance system," he remarks, "is undoubted. It would entail less red-tape and friction. It would be more consistent with our habits of thought and life. It would be regularised, though voluntary." To all these arguments the inevitable reply is a question, "Why?" Why, to take one instance only, should the dispensary system be more consistent with English than with German habits of thought and life? Because it was tried and broke down in Germany? The hospitals of the Barmhertzige Bruder in Austria and in Germany are still provident dispensaries to a large extent, and yet, for all the good work they undoubtedly do, they can scarcely be said to have dealt with the problem of hospital relief in a thorough manner. How much headway has the provident dispensary system made in England up to the present time? Ask the average general practitioner—not the man who finds time to leave his practice to attend meetings of the Association in London, and who opposes any scheme of relief which he thinks likely to influence his income adversely, but the man who works in a poor-class neighbourhood, and who blesses the existence of any organisation, hospital or otherwise, in his district because it enables him to deal with the mass of his poorer patients in a much more thorough and successful manner than he would otherwise be able to do—ask such a practitioner what difference the establishment of a provident dispensary in his district has made to his poorer private patients. The fact is, no provident dispensary can exist without a hospital, and so long as the *clientèle* of these dispensaries remains as small as it is at present they will not be able to provide hospitals sufficiently large and sufficiently well equipped to deal with the hospital population in their own districts.

### THE COMPULSORY INSURANCE SCHEME.

Dr. Loch seems to admit that there is something wanting in the dispensary system, for he is careful to state that his favourable view of it is not by implication an opposition to a contributory scheme of invalidity insurance for long-continued or permanent disablement. But invalidity insurance on a contributory basis, as has been conclusively proved in the case of Germany, means the practical abolition of the dispensary system, provided it is a compulsory scheme. And there is no reason to believe that the community favours a voluntary contributive scheme such as it already possesses in the shape of the various friendly and other societies, unions, and insurance companies. We have no wish to criticise Dr. Loch's criticisms in full; we merely allude to some of the arguments which may be used against the pros and cons with which he has dealt in his interesting paper. What is specially wanted at present is a summary of the methods existing in other countries. We have lately heard much of the German example, but no one seems to have thought it worth while to investigate the various systems that prevail in Austria and in Italy, which, by comparison with those in this country and in Germany, are instructive and interesting. It is only by a comparison of the manner in which these various systems have worked so far that we can arrive at any definite conclusions. To state that one or other of them is unsuitable for adoption in this country on purely theoretical grounds is simply begging the question, which should be discussed in all its bearings and without undue consideration to any one aspect of it—such as, for instance, the remuneration of medical men or the independent existence of semi-public hospitals.

### "WORKERS' HOSPITAL FUNDS."

Under this heading a paragraph appeared on page 534 of our issue of July 30 which briefly described the rules, aims, and organisation of a Workers' Fund, then newly started at Cambridge. There we stated that "we looked forward to seeing the hope of its authors at Cambridge realised, that £1,000 will be raised at the end of the first year." The results of the first quarter's work have justified this expectation. After three months' activity, as the secretary pointed out at a recent meeting, sixty-two centres of collection have been founded; and, it appears that an even greater number might have been established if the employers had been as easy of access as their workpeople. Without the permission of these retiring gentlemen it is difficult to find an opportunity of explaining the scheme to the workers, and that this explanation is not a lengthy one is shown by the fact that at one place four meetings were addressed in two hours. The indifference of a few employers is therefore unreasonable, as indeed is proved by those of them who have come forward already. It is through the latter's courtesy that, as Mr. Cant, the treasurer, pointed out, £166 has been raised this quarter, which already guarantees the better half of the £1,000 per annum that was aimed at originally. Mr. Dyball prophesied that this sum would approach £800 before long. Addenbrooke's requires an income of £10,000 a year, and the growing population of Cambridge, as Colonel T. W. Harding, President of the Workers' Society and Chairman of the Hospital's Finance Committee, pointed out, is making extension imminent. A noticeable point in the scheme, as we mentioned previously, is the power of the subscribers to allocate three-tenths of their contribution to the Convalescent Homes, for which they can receive recommendations in proportion. It is very satisfactory to find that this idea of the Workers' Fund, which hitherto has been confined to the industrial population of the North, can flourish successfully, if a district is added, in the retail businesses of county towns in the South. Again we recommend the capital example of Cambridge to other towns of a similar type.

### THIS WEEK'S DRUG MARKET.

Business has been somewhat quieter this week, and few noteworthy alterations in price have taken place. Cocaine hydrochloride is again rather dearer, and a further small advance in price is not altogether improbable. Following on the reduction in the price of quicksilver, makers of mercurial salts have reduced the price of calomel, corrosive sublimate, etc., by one penny per lb. Sugar of milk is tending dearer. Gum benzoin is fetching higher prices. Seidlitz powder and tartarated soda are both slightly dearer again. Opium is tending rather dearer, but morphine and codeine are unchanged in price.

